# Environmental Gerontology During COVID-19: Aging in Place Since the Pandemic Onset

**DOI:** 10.1093/geroni/igab046.1228

**Published:** 2021-12-17

**Authors:** Melissa Cannon, Jessica Finlay, Graham Rowles

## Abstract

The COVID-19 pandemic is fundamentally changing neighborhood landscapes as we shelter in place and adjust our lifestyles. To age-in-place is to live in one’s home and/or community “safely, independently, and comfortably.” The ability to age-in-place is a public health priority for all, regardless of income or health status, and requires a variety of community resources to be sustainable. Since the pandemic onset, access to neighborhood resources was limited to reduce transmission risks. Changes to economic arrangements and socio-spatial norms have profoundly impacted daily life, though how these influence health and well-being is largely unknown. It is likely that these effects may vary in different communities and contexts; for example, neighborhoods that are able to self-organize to safely provide social support and resources may fare better. This symposium brings together cutting-edge studies in urban and rural U.S. places to explicate how the pandemic is transforming aging-in-place experiences and perspectives. The first presentation shows how rapidly community-based services have adjusted operations to meet the needs of their communities. The second presentation explores strategies to provide social support in rural communities. The third presentation highlights the social health needs of a subset of older adults who had not formed friendships with their neighbors. Together, these studies suggest that close examinations of aging-in-place conditions and mechanisms from organizational, socio-spatial, and social network perspectives are evermore important amid a pandemic. We discuss the implications of these empirical findings in relation to emerging theories within environmental gerontology.

